# Enhanced Enzymatic Performance of Immobilized *Pseudomonas fluorescens* Lipase on ZIF-8@ZIF-67 and Its Application to the Synthesis of Neryl Acetate with Transesterification Reaction

**DOI:** 10.3390/molecules29122922

**Published:** 2024-06-19

**Authors:** Qi Wang, Jian Xiong, Hanghang Xu, Wenyuan Sun, Xiaoxu Pan, Shixin Cui, Siting Lv, Yinling Zhang

**Affiliations:** School of Chemistry and Chemical Engineering, Henan University of Science and Technology, Luoyang 471023, China

**Keywords:** lipase, ZIF-8@ZIF-67, immobilization, neryl acetate, transesterification reaction

## Abstract

In this study, hybrid skeleton material ZIF-8@ZIF-67 was synthesized by the epitaxial growth method and then was utilized as a carrier for encapsulating *Pseudomonas fluorescens* lipase (PFL) through the co-precipitation method, resulting in the preparation of immobilized lipase (PFL@ZIF-8@ZIF-67). Subsequently, it was further treated with glutaraldehyde to improve protein immobilization yield. Under optimal immobilization conditions, the specific hydrolytic activity of PFL@ZIF-8@ZIF-67 was 20.4 times higher than that of the free PFL. The prepared biocatalyst was characterized and analyzed by scanning electron microscopy (SEM), X-ray diffraction (XRD), and Fourier transform infrared (FT-IR). Additionally, the thermal stability of PFL@ZIF-8@ZIF-67 at 50 °C was significantly improved compared to the free PFL. After 7 weeks at room temperature, PFL@ZIF-8@ZIF-67 retained 78% of the transesterification activity, while the free enzyme was only 29%. Finally, PFL@ZIF-8@ZIF-67 was applied to the neryl acetate preparation in a solvent-free system, and the yield of neryl acetate reached 99% after 3 h of reaction. After 10 repetitions, the yields of neryl acetate catalyzed by PFL@ZIF-8@ZIF-67 and the free PFL were 80% and 43%, respectively.

## 1. Introduction

Lipase (EC 3.1.1.3), an important industrial enzyme that catalyzes reactions such as esterification, transesterification, enantioselective hydrolysis, and kinetic resolution of racemates, is widely used in the food, energy, detergent, pharmaceutical, leather, textile, cosmetics, and paper industries [1,2,3,4,5]. Lipase is a very special enzyme, having a peculiar mechanism of action called interfacial activation [6]. The lipase activity center is flexible and covered by a relatively conserved polypeptide chain structure called a ‘lid’. The lipase lid has a hydrophilic external surface and a hydrophobic internal surface. In homogenous media, a large percentage of most lipase molecules have their active centers covered by the lid, which may isolate them from the reaction medium (closed form) [7]. In the presence of a hydrophobic surface, the enzyme becomes adsorbed on it, the lid structure will be opened, and its active center will be exposed. In aqueous and homogeneous systems, this open form is unstable [8]. The relatively high cost of lipase as a catalyst, its unstable structure, and the difficulty of recovery have limited its further use in industrial applications [9]. To overcome the many drawbacks of free lipase, enzyme immobilization techniques have been investigated. In comparison to the free enzyme, the immobilized enzyme has several advantages, such as easier separation and recycling, higher activity, more stable enzymes, more resistance to various environmental changes, non-toxicity and biocompatibility, or coupling with enzyme purification, making it suitable for more complex reaction environments and a variety of reactor configurations [3,10,11,12]. However, some lipases react with hydrophobic matrixes or other lipase molecules during the immobilization process, resulting in lipase inactivation. In general, the immobilized enzyme properties are affected by carrier materials, immobilization techniques, and enzyme properties [3,13]. The selections of the optimum carrier and the immobilization technique are very important for enzyme immobilization success.

Metal organic frameworks (MOFs) are excellent carriers for immobilized lipases because of their porosity, high specific surface areas, modifiable structure, adjustable pore channels, designable frameworks, and easy functionalization [14,15]. These studies have shown that embedding lipases in MOFs to construct immobilized enzyme systems is more conducive to improving the inherent fragility of lipases and, at the same time, to endowing MOFs with new biological functions [16]. To further improve the performance of single MOFs, more and more researchers have synthesized MOF hybrid frameworks (MOF@MOF) with controlled components, complex morphology, and structure [17]. MOF@MOF is a mixed framework formed by various chemical interactions between different MOFs that often has a core shell or a layered heterostructure. Due to the good synergistic effect among different MOFs materials, these hybrid frameworks often exhibit multifunctional properties and are widely used in wastewater treatment, chemical catalysis, and sensor detection [18,19,20]. Zeolitic imidazolate frameworks (ZIFs) are the most commonly studied materials for MOF@MOF. Research in biochemistry is mainly focused on biosensor detection. Xiao et al. [21] prepared ZIF-67@ZIF-8-P by pre-introducing P into ZIF-67@ZIF-8 through a selective phosphorylation reaction, and a novel CoP@C/NCS detector was constructed using it as a precursor. The detector showed good performance in the detection of dopamine (DA) with a sensitivity of 9.4 µA/µM and a lower detection limit of 0.03 µM. Furthermore, the electrocatalytic oxidation performance of CoP@C/NCS for DA was superior to that of CoP/C prepared on the basis of a single ZIF-67. Cao et al. [22] prepared a PCN-222@ZIF-8 sensor using polyvinylpyrrolidone (PVP) to modify ZIF-8, after which the modified ZIF-8 was coated on PCN-222, and the results showed that the PCN-222@ZIF-8 sensor was more selective for catechol and 10 times more sensitive than the single PCN-222 sensor; in addition, PCN-222@ZIF-8 had good solvent adaptability to overcome the disadvantages of natural horseradish peroxidase.

Neryl acetate (C_12_H_20_O_2_) is a compound with a light neroli fragrance, and its aroma can be comparable to more than 20 types of essential oil, including citronella oil, lemon eucalyptus oil, geranium oil, etc. It is widely used in the food industry as an edible flavor [23]. In the pharmaceutical field, neryl acetate has a positive effect on the treatment of acne vulgaris [24]. In the feed industry, the EU Food Safety Authority has approved neryl acetate as a safe feed flavoring for animals. In addition, certain concentrations of neryl acetate have insecticidal activity and are widely used to control corn weevil [25]. In the study of neryl acetate preparation, its efficient synthesis using the lipase-catalyzed transesterification reaction is an important direction of current research [26,27].

In this study, *Pseudomonas fluorescens* lipase (PFL) was immobilized by the co-precipitation method using ZIF-8@ZIF-67 as the carrier. The immobilized lipase, PFL@ZIF-8@ZIF-67, was prepared by optimizing the immobilization conditions. The storage stability and thermal stability were investigated. The prepared PFL@ZIF-8@ZIF-67 was applied to a solvent-free system for the preparation of neryl acetate by transesterification of nerol with vinyl acetate, the reusability of PFL@ZIF-8@ZIF-67 was investigated, and the reaction conditions were optimized for the efficient synthesis of neryl acetate.

## 2. Results and Discussion

### 2.1. Optimization of the Preparation Conditions for PFL@ZIF-8@ZIF-67

#### 2.1.1. Amount of ZIF-8

**Figure 1:**
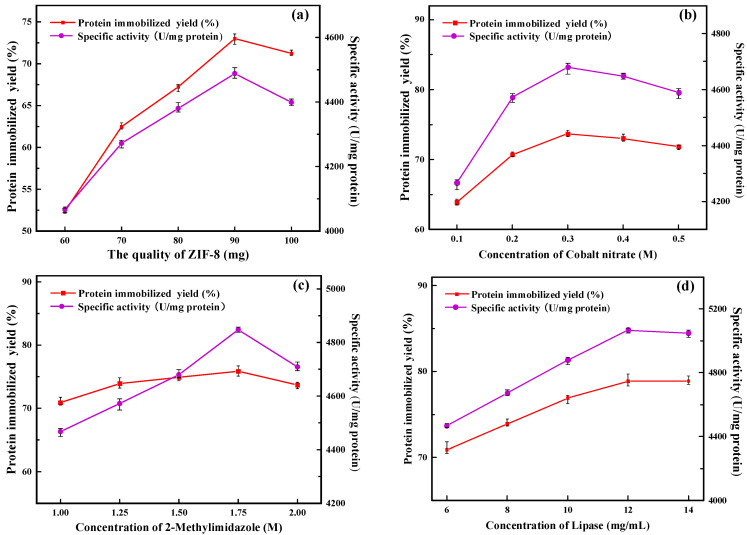

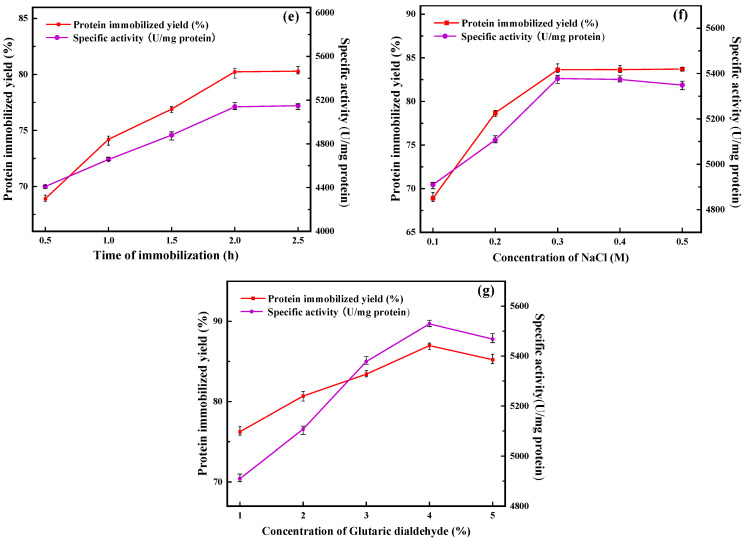
Optimization of the preparation conditions for PFL@ZIF-8@ZIF-67: (**a**) amount of ZIF-8; (**b**) concentration of cobalt nitrate; (**c**) concentration of 2-methylimidazole; (**d**) concentration of lipase; (**e**) immobilization time; (**f**) concentration of sodium chloride; (**g**) concentration of glutaraldehyde.

In this study, ZIF-8 was used as seed crystal and ZIF-67 as secondary growth crystal for the preparation of immobilized lipase. ZIF-67 grew freely on the surface of ZIF-8, accompanied by the immobilization of PFL, and finally formed PFL@ZIF-8@ZIF-67. As shown in Figure 1a, with the amount of ZIF-8 increasing, the protein immobilization yield and the specific activity of PFL@ZIF-8@ZIF-67 first increased and then decreased. When the mass of ZIF-8 was 90 mg, the protein immobilization yield and the specific activity of PFL@ZIF-8@ZIF-67 were the highest. This is because ZIF-8 acts as a seed crystal, and as the amount increases, more and more ZIF-8 is wrapped by ZIF-67, so the amount of ZIF-8@ZIF-67 in the preparation system increases, which is more favorable for lipase encapsulation [28]. When the amount of ZIF-8 exceeded 90 mg, part of ZIF-8 could not be fully wrapped by ZIF-67 and aggregated in the preparation system, increasing mass transfer resistance and affected lipase encapsulation, and then affected protein immobilization yield and specific activity [29]. In subsequent experiments, 90 mg of ZIF-8 was finally chosen as the amount of ZIF-8.

#### 2.1.2. Concentration of Cobalt Nitrate

As shown in Figure 1b, the protein immobilization yield and the specific activity of PFL@ZIF-8@ZIF-67 first increased and then decreased as the concentration of cobalt nitrate kept increasing. When the concentration of cobalt nitrate was less than 0.3 M, it was favorable for ZIF-8@ZIF-67 to form a more complete crystal structure with an increasing concentration of cobalt nitrate, and the lipase was continuously encapsulated inside the carrier. As the cobalt nitrate concentration was further increased, the protein immobilization yield and specific activity of PFL@ZIF-8@ZIF-67 both showed decreases. This is because excess metal ions can have an inhibitory effect on lipase [26], and as the concentration of cobalt nitrate increases, Co^2+^ in solution inhibits the activity of lipase through a non-competitive effect. The cobalt nitrate concentration was finally chosen as 0.3 M for the following experiments.

#### 2.1.3. Concentration of 2-Methylimidazole

As seen in Figure 1c, the protein immobilization yield and the specific activity of PFL@ZIF-8@ZIF-67 improved continuously with the increase in 2-methylimidazole concentration in the concentration range of 1.00 M to 1.75 M. This process is conducive to the formation of a more complete crystal structure of ZIF-8 @ ZIF-67, and the lipase is continuously encapsulated in the carrier [30]. The protein immobilization yield and specific activity of PFL@ZIF-8@ZIF-67 decreased as the concentration of 2-methylimidazole was further increased. Enzymes, as biocatalysts, are susceptible to microenvironmental influences such as heavy metals, acids and bases, and organic compounds. The 2-methylimidazole solution is alkaline, and as its concentration increases, the pH of the solution increases, causing the enzyme’s catalytic action to be outside the optimal pH range [31], which in turn decreases its specific activity. The final concentration of 2-methylimidazole for the following experiments was chosen as 1.75 M.

#### 2.1.4. Concentration of Lipase

The concentration of enzymes is one of the main factors that affect the immobilized enzyme loading in the immobilization system, and the amount of enzyme loading directly affects the specific activity of the immobilized enzyme. Choosing a suitable enzyme concentration not only can maximize the immobilized protein and the activity of the immobilized enzyme but also can avoid wasting lipase and save money. The protein immobilization yield and the specific activity of PFL@ZIF-8@ZIF-67 continued to increase as the concentration of PFL increased (Figure 1d). This indicates that ZIF-8@ZIF-67 has a high loading of lipase, in which more and more lipase is encapsulated in the carrier. The highest PFL@ZIF-8@ZIF-67 protein immobilization yield and the specific activity were achieved when the lipase concentration was 12 mg/mL. The concentration of lipase is too high, and agglomeration may occur, which will affect its specific activity [32]. The amount of PFL@ZIF-8@ZIF-67 for the following experiments was finally selected to be 12 mg/mL based on a combination of catalytic activity and economic efficiency.

#### 2.1.5. Immobilization Time

In the process of preparing immobilized enzymes, the immobilization time has a great influence on the protein immobilization yield and the specific activity of the final immobilized enzymes. On the one hand, if the immobilization time is too short, the lipase cannot bind well to the carrier, making the immobilization yield and the specific activity lower; on the other hand, if the immobilization time is too long, the long-term mechanical stirring will damage the advanced structure of the enzyme to some extent, making the final immobilized enzyme specific activity lower. It can be seen in Figure 1e that the protein immobilization yield and the specific activity of PFL@ZIF-8@ZIF-67 increased with increasing immobilization time. When the immobilization time exceeded 2 h, the increase in immobilization yield and specific activity leveled off, and the optimal immobilization time was reached at this time. This is because in the early stage of immobilization, the binding of the enzyme to the support is carried out at a high yield. However, with time, the yield of lipase encapsulation by the support becomes saturated. Scanning electron micrographs of immobilized enzymes with different immobilization times (2 h and 2.5 h) were compared (Figure S1, Supplementary Material). It was found that the structure of the immobilized enzyme was not significantly altered. Therefore, 2 h was chosen as the immobilization time for subsequent experiments.

#### 2.1.6. Concentration of Sodium Chloride (NaCl)

With the increase in NaCl concentration, the protein immobilization yield of PFL@ZIF-8@ZIF-67 continuously increased and finally stabilized (Figure 1f). The specific activity of PFL@ZIF-8@ZIF-67 was highest when the NaCl concentration was 0.3 M. This is because the moderate concentration of NaCl can cause ZIF-8@ZIF-67 to form more microporous structures and promote the connection between nanoparticles, which is more conducive to the encapsulation of lipase. Also, the appropriate concentration of NaCl can be used as a protective agent to improve the structural solidity of ZIF-8@ZIF-67 and avoid the collapse of nanostructures during the synthesis of nanostructures [33]. When the NaCl concentration exceeded 0.3 M, the specific activity of PFL@ZIF-8@ZIF-67 appeared to be decreased slightly, which might be due to the fact that excess NaCl would affect the active site of lipase, resulting in the decrease in the specific activity. Taking this into account, 0.3 M NaCl concentration was finally chosen for the following experiments.

#### 2.1.7. Concentration of Glutaraldehyde

In this study, the immobilization of PFL occurred in two steps. The first step was the encapsulation of the lipase on the support, and the second step was the covalent reactions between the glutaraldehyde groups and the enzyme Schiff-base formation between the primary amino groups of enzymes (terminal amino groups or ε-amino group of lysine residues) [34]. Figure 1g shows the curves of the protein immobilization yield and the specific activity with the concentration of glutaraldehyde. It can be seen from the figure that the protein immobilization yield and the specific activity of PFL@ZIF-8@ZIF-67 were the highest when the glutaraldehyde concentration was 4%. Glutaraldehyde, as a crosslinking agent, has good polymerization of protein molecules, and the appropriate amount of glutaraldehyde will improve the protein immobilization yield and activity of PFL@ZIF-8@ZIF-67. When the concentration of glutaraldehyde in the system was low, the number of aldehyde groups in the reaction system was small, and intermolecular crosslinking could easily occur, thus maintaining the spatial configuration of protein molecules and improving the immobilization yield and activity of lipase [35]. When the concentration of glutaraldehyde exceeded 4%, the protein immobilization yield and activity decreased because glutaraldehyde was both a crosslinking agent for immobilization reactions and a denaturant that can inactivate the enzyme. At this time, the reaction system was prone to intramolecular crosslinking and multi-point binding of the aldehyde group to the amino group on the enzyme protein molecule, leading to changes in the advanced structure of the enzyme protein and destroying part of the active site of the enzyme, thus reducing activity [36]. Moreover, distortion of the secondary structure of lipase directly by high glutaraldehyde concentration in crosslinking treatment resulted in greater inactivation of lipase [34]. In the end, a 4% glutaraldehyde concentration was chosen.

Following these optimal conditions, there was a 20.4-times increase in the specific activity of PFL@ZIF-8@ZIF-67 (5529 U/mg protein) compared to the free PFL (271 U/mg protein). Lipase has a portable component termed as the ‘lid’, which covers the active catalytic center and controls the flow of substrate to the active site of the enzyme. The secondary structure associated with PFL has the potential to be altered by the structure of ZIF-8@ZIF-67. For a period of time, the lid is accessible to the substrate, leading to improvement in lipase activity due to the facility of passage [32].

### 2.2. Characterization and Kinetics Parameter of PFL@ZIF-8@ZIF-67

#### 2.2.1. SEM Analysis

**Figure 2:**
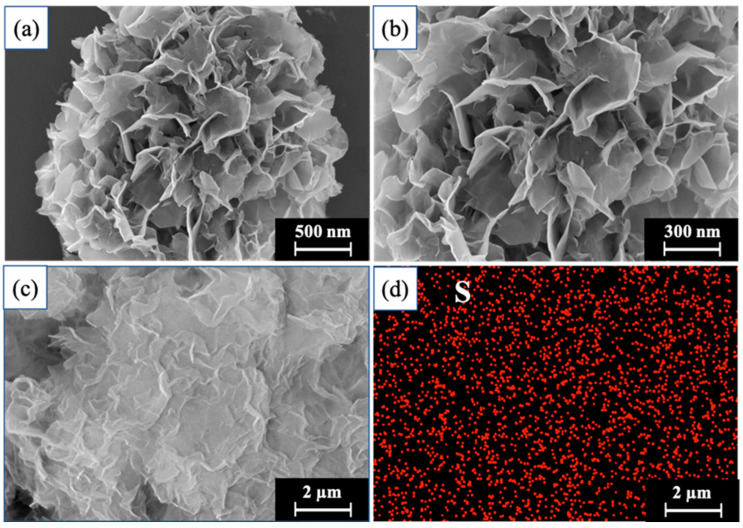
SEM analysis of PFL@ZIF-8@ZIF-67: (**a**–**c**) SEM images; (**d**) the mapping of S elements on a local scale.

As can be seen from the electron micrographs (Figure 2a,b), the PFL@ZIF-8@ZIF-67 prepared under the optimized conditions had an overall “peony flower” shape, with more contact space between the enzyme protein and the spatial folds of the “flower petals” and more binding sites. This was very favorable for the enzyme protein molecules to be immobilized on ZIF-8@ZIF-67. Since lipase contains disulfide bonds, the elemental mapping of S elements on the ZIF-8@ZIF-67 carrier was investigated to determine the distribution of lipase on the carrier. As shown in Figure 2c,d, the element S was evenly distributed on the surface of the carrier, demonstrating that the lipase was immobilized on the ZIF-8@ZIF-67 carrier.

#### 2.2.2. XRD Analysis

**Figure 3:**
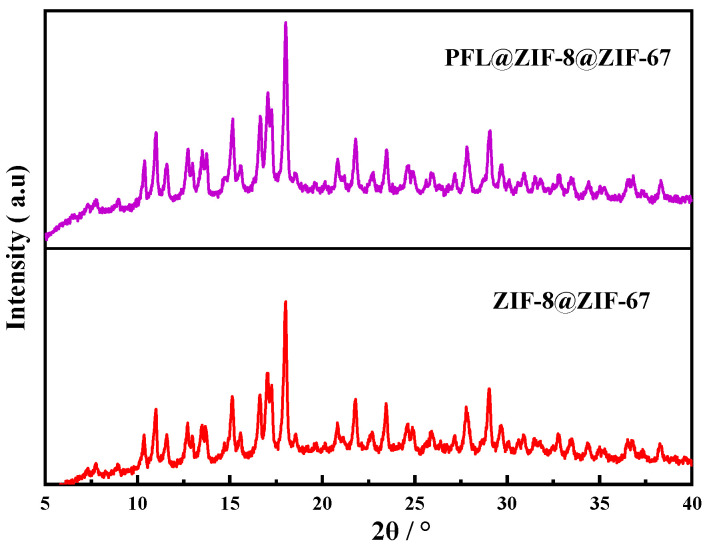
XRD comparison of ZIF-8@ZIF-67 and PFL@ZIF-8@ZIF-67.

The XRD patterns of PFL@ZIF-8@ZIF-67 and ZIF-8@ZIF-67 are shown in Figure 3. Both crystals had strong peaks at 2θ of 10.98°, 14.83°, 16.75°, 18.20°, and 21.87°, consistent with the previously reported ZIF-8@ZIF-67 diffraction [37]. This behavior indicated that the immobilization of the enzyme did not affect the crystal structure of ZIF-8@ZIF-67.

#### 2.2.3. FT-IR Analysis Analysis

**Figure 4:**
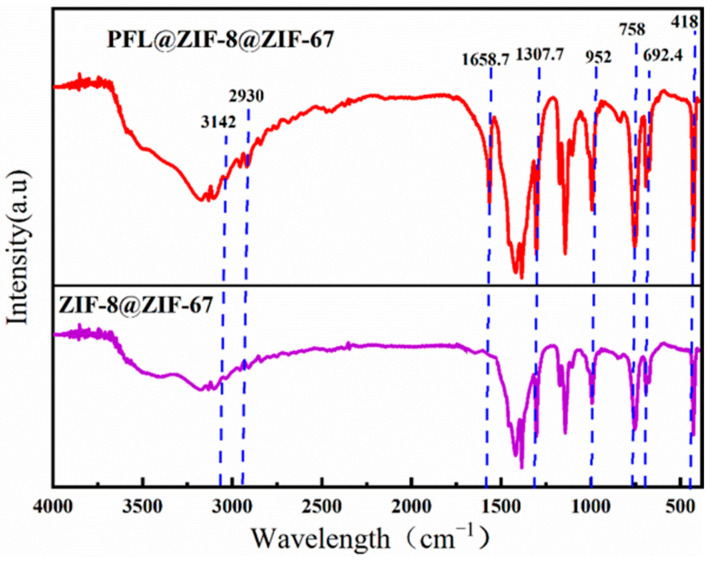
FT-IR comparison of ZIF-8@ZIF-67 and PFL@ZIF-8@ZIF-67.

Figure 4 shows the IR spectra of ZIF-8@ZIF-67 and PFL@ZIF-8@ZIF-67, in which the representative Zn-N stretching band of ZIF-8@ZIF-67 is observed at approximately 418 cm^−1^. The strong adsorption bands at 758 cm^−1^ and 692. 4 cm^−1^ belonged to the out-of-plane bending of the Hmim ring, while those at 952.8 cm^−1^ and 1307.7 cm^−1^ are from in-plane bending. The characteristic peaks of imidazole ligands were strong in these regions, which indicated that organic ligands participated in the reaction. In addition, two weak peaks at 2930 cm^−1^ and 3124 cm^−1^ can be attributed to the aliphatic and aromatic CeH stretching of Hmim, respectively, which was consistent with the structure of ZIF-8@ZIF-67 reported in the literature, demonstrating the successful synthesis of ZIF-8@ZIF-67 in this experiment. After the immobilization of lipase, the IR spectrum of PFL@ZIF-8@ZIF-67 showed the characteristic amide I band of lipase at 1658.7 cm^−1^, corresponding to the NeH bending mode, confirming that lipase was successfully immobilized on ZIF-8@ZIF-67 [38,39].

#### 2.2.4. Michaelis–Menten Kinetic Parameters of PFL@ZIF-8@ZIF-67

**Figure 5:**
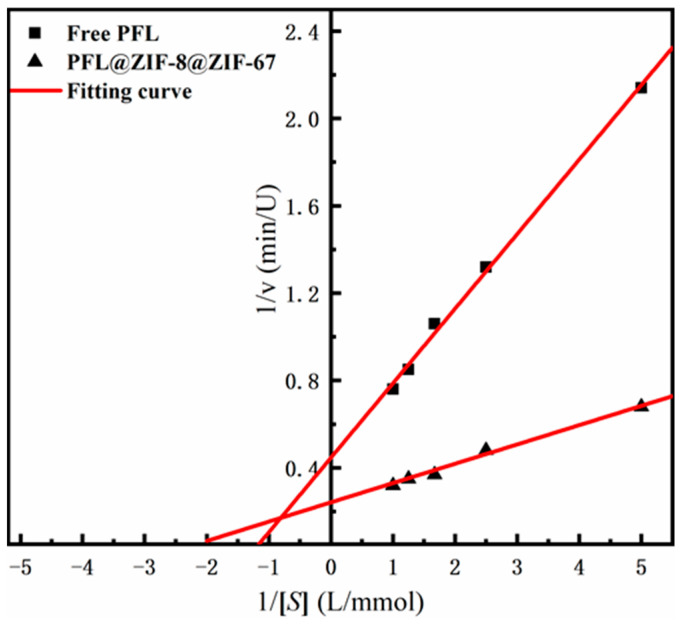
Lineweaver–Burk plot of ZIF-8@ZIF-67 and PFL@ZIF-8@ZIF-67.

The kinetic constants of the enzymes, including K_m_ and V_max_ of PFL@ZIF-8@ZIF-67 and the free PFL, were obtained by linear regression of different substrate concentrations and initial reaction velocity catalyzed by the enzymes (Figure 5). The V_max_ value of PFL@ZIF-8@ZIF-67 was 4.13 Mm/min, which was greater than the free PFL (2.24 mM/min). The significant increase in V_max_ could indicate that the lipase immobilized on ZIF-8@ZIF-67 has a higher catalytic efficiency than the free enzyme, which is related to the activation of the lipase [40]. The K_m_ value of the immobilized lipase (0.36 mM) was lower than that of the free lipase (0.76 mM), indicating that the affinity of the enzyme to the substrate was improved after immobilization. Some studies indicated that the decrease in K_m_ was related to the hydrophobic support and the open conformation of lipase [41,42].

### 2.3. Application of PFL@ZIF-8@ZIF-67 in the Synthesis of Neryl Acetate

#### 2.3.1. Effect of Immobilized Lipase Amount 

**Figure 6:**
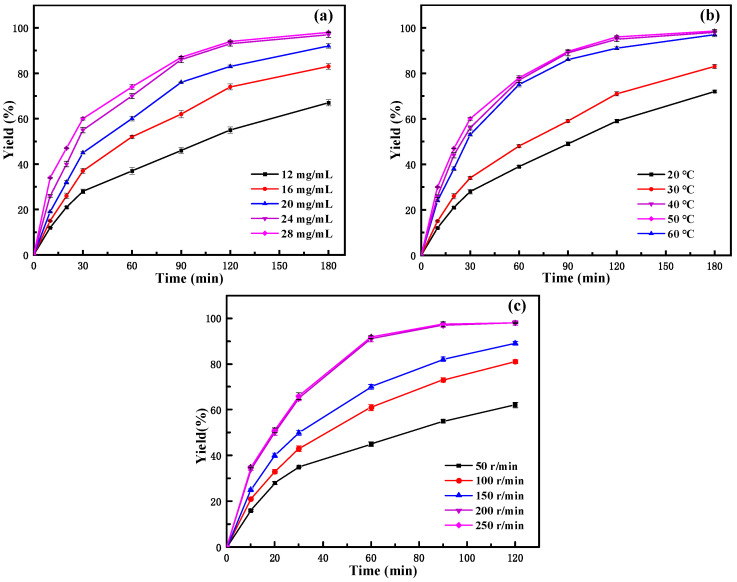
Effect of reaction conditions on the yield of neryl acetate: (**a**) immobilized lipase amount; (**b**) temperature; (**c**) rotating speed.

From the influence of the amount of immobilized enzyme in Figure 6a, it can be seen that, at the same reaction time, with the increase in the immobilized enzyme amount, the yield gradually increased, especially in the initial stage of the reaction (before 30 min). At the same time, it could be seen from the figure that the yield of neryl acetate obviously increased at the initial stage of the reaction. After 2 h of reaction, the growth tends to be stable. In a certain range of enzyme concentrations, the higher the enzyme concentration, the higher the probability of enzyme contact with the substrate, and the faster the catalytic rate. The amount of enzyme is related not only to the reaction speed but also has an important impact on the production cost of products. Taking into account the factors of yield and economic benefit, 24 mg/mL of immobilized lipase was selected as the catalyst addition.

#### 2.3.2. Effect of Reaction Temperature

Temperature is an important parameter that affects enzyme-catalyzed reactions, and choosing the appropriate reaction temperature not only helps to prolong the life of the enzyme but also increases the rate of the reaction [43]. From the effect of the reaction temperature (Figure 6b), it could be seen that the yield of neryl acetate increases with increasing temperature in the experimental temperature range studied. With increasing temperature, the initial reaction speed increased and then decreased, while the yield of neryl acetate continuously increased and finally stabilized. The initial reaction rate and yield were highest at 50 °C. When the temperature reached 60 °C, the initial rate decreased, and the final conversion rate was similar to that at 50 °C. The results indicated that the optimal reaction temperature for the immobilized enzyme to catalyze the reaction was 50 °C. Compared to the optimal reaction temperature of 40 °C for the free enzyme [27], it increased by 10 °C. This also indicated that the thermal stability of the immobilized enzyme was greatly improved compared to that of the free enzyme. This is because the rigidity of the enzyme increases after it is immobilized on the carrier. To make it more active and combine with the substrate molecules, it is necessary to increase the temperature to increase its flexibility and improve the contact of the enzyme with the substrate. Therefore, the catalytic reaction temperature of immobilized lipase is higher than that of the free lipase.

#### 2.3.3. Effect of Rotating Speed

From the effect of the reaction speed as shown in Figure 6c, it can be seen that at the same reaction time, the yield increases with increasing reaction speed. Also, it can be seen that when the reaction speed is greater than 200 r/min, the yield growth rate tends to be basically stable, which indicates that when the reaction speed reaches 200 r/min, the external diffusion restriction in the reaction system is largely eliminated. The immobilized enzyme reaction system was a heterogeneous reaction system, so the combination of enzyme and substrate molecules was restricted by external diffusion, which affects the catalytic efficiency of the enzyme in the organic phase [44]. The influence of external diffusion could be reduced or even eliminated by studying the influence of the shaking table speed. The literature indicated that rotation speed increases the diffusion of the reactants to the interfacial area of the enzyme and, thus, their availability for the reaction by reducing external mass transfer limitations [45,46]. Considering that the increase in product yield was not obvious at higher speeds, 200 r/min was chosen as the reaction speed to reduce the energy consumption as much as possible without affecting the mass transfer effect. After the above optimization, the yield of neryl acetate could reach over 99% after 3 h of reaction. This was a significant improvement on our previous study [27], and although the final yield of neryl acetate was almost the same, the reaction time was greatly reduced. In contrast to other literature, Zong et al. [47] successfully produced neryl acetate by biosynthesis using recombinant *Escherichia coli*. Although the maximum production of neryl acetate reached 11.712 ± 0.653 mg/L by increasing pyruvate, the process of synthesizing neryl acetate by this method was too complicated, and large-scale industrial production was difficult. Therefore, the synthesis of neryl acetate using PFL@ZIF-8@ZIF-67 has distinct advantages, which may be helpful for the efficient production of neryl acetate in the future.

### 2.4. Stability of PFL@ZIF-8@ZIF-67

#### 2.4.1. Reusability of PFL@ZIF-8@ZIF-67

**Figure 7:**
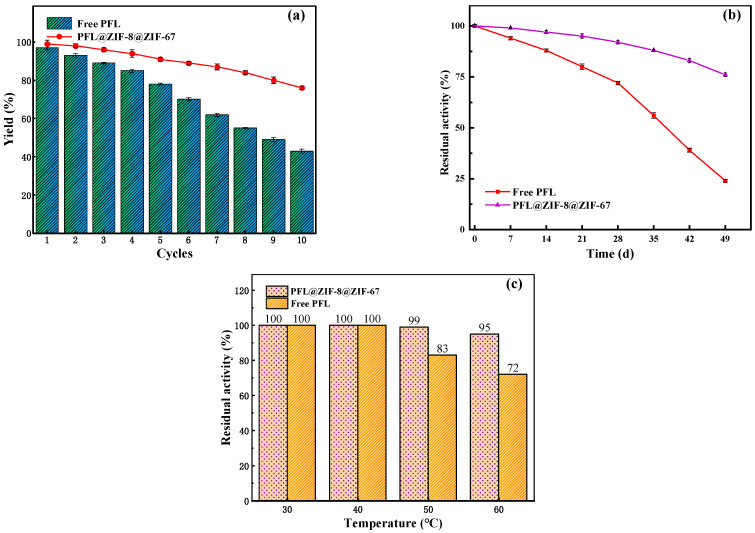
Stability of reaction conditions on the yield of neryl acetate: (**a**) reusability; (**b**) storage stability; (**c**) thermal stability.

The reusability of enzymes is an important evaluating indicator in practical applications. Figure 7a shows the yield of neryl acetate with immobilized enzyme and the free enzyme after 10 cycles. It was observed that the yield decreased after each cycle. The lower enzymatic activity was attributed to protein denaturation and leakage of protein from the carrier. After 10 reuses, the yield catalyzed by the free PFL was only 43%, whereas that by PFL@ZIF-8@ZIF-67 was 80%. The results illustrated that the immobilized enzyme had higher reusability than the original PFL. The improved recyclability of PFL@ZIF-8@ZIF-67 was mainly caused by the prevention of enzyme desorption, since the MOF@MOF framework and the lipase molecule formed a strong interaction [48]. These data confirmed that the minimal decrease in activity of immobilized lipases was absent of enzyme leakage, and the enzyme retained its conformation during 10 repetitive cycles. Such a decrease in reusability can be attributed to the long period of time that the enzyme was dipped in the reactants, especially the product (neryl acetate). The accumulation of these components inside the biocatalyst particle can produce enzyme inhibition/inactivation. The impact of this problem can be solved by applying ultrasound [49,50] or using a solvent with moderate polarity that is able to solubilize efficiently all the reactants [51]. Also, the use of very hydrophobic supports to immobilize the lipase could be chosen in order to avoid the adsorption of hydrophilic compounds [52].

#### 2.4.2. Storage Stability of PFL@ZIF-8@ZIF-67

Generally, the enzymatic activity decreased gradually with the storage time. The storage stabilities of PFL@ZIF-8@ZIF-67 and the free PFL were examined in the closed conical flask at room temperature for 49 days with a 7-day interval. The results are shown in Figure 7b. After seven weeks of storage, PFL@ZIF-8@ZIF-67 and the free lipase displayed that the residual transesterification activities were 79% and 28%, respectively. The increased storage stability might be due to the protective effect provided by MOF@MOF encapsulation around enzyme molecules, which resulted in minimal distortion caused by environment on the active sites of the enzyme [53].

#### 2.4.3. Thermal Stability of PFL@ZIF-8@ZIF-67

Thermal stability is very important for an immobilization method. The free lipase and PFL@ZIF-8@ZIF-67 were incubated at different temperatures for 6 h and lipase activity was measured, as shown in Figure 7c. When the temperature was less than 40 °C, the transesterification activities of the free enzyme and the immobilized enzyme did not decrease. When the temperature was higher than 40 °C, the transesterification activity of PFL@ZIF-8@ZIF-67 decreased, but the degree of decrease was lower than that of the free PFL. At 60 °C, the residual activities of PFL@ZIF-8@ZIF-67 and the free PFL retained 95% and 72%, respectively. The interactions between lipase and MOF@MOF during the immobilization process can be explained by the enzyme’s protection of its tertiary structure [54].

## 3. Materials and Methods

### 3.1. Experimental Material

The lipase powder used in the experiments was derived from *Pseudomonas fluorescens* and purchased from Amano Enzyme Ltd. (Nagoya, Japan). Bovine serum albumin, nerol, p-nitrophenol (p-NP), and p-nitrophenol butyrate (p-NPB) were obtained from Sigma-Aldrich (St. Louis, MO, USA). Standard neryl acetate was supplied by Tokyo Chemical Industry Co. Ltd. (Tokyo, Japan). Other chemicals and solvents are commercially available analytical grade reagents. All the chemicals and enzymes used have not been further treated.

### 3.2. Preparation of PFL@ZIF-8@ZIF-67

PFL@ZIF-8@ZIF-67 immobilized lipase was prepared by the co-precipitation method, and the effects of various factors on PFL@ZIF-8@ZIF-67 during the preparation process were investigated. The specific experimental steps were as follows: zinc nitrate hexahydrate solution (0.8 mL, 0.5 M) and 2-methylimidazole solution (8 mL, 1.25 M) were ultrasonicated (40 kHz, 5 min) to make them well dispersed; the two solutions were mixed, and the reaction was stirred at room temperature (500 r/min, 1 h), and the white precipitates were collected by centrifugation (8500 r/min, 10 min) and washed with deionized water for 3 times. Then, it was dried in a vacuum drying oven to constant weight, and finally, ZIF-8 was prepared.

ZIF-8 suspension was prepared by adding 90 mg of the above prepared ZIF-8 to 8.8 mL of deionized water and ultrasonication (40 kHz, 5 min) to disperse it; PFL solution (4 mL, 6 mg/mL), cobalt nitrate hexahydrate solution (0.8 mL, 0.3 M), 2-methylimidazole solution (8 mL, 1.75 M), and NaCl solution (8 mL, 0.3 M) were sonicated (40 kHz, 5 min) to make them well dispersed. The above 4 solutions were added simultaneously to the ZIF-8 suspension, and the reaction was stirred at room temperature (500 r/min, 1 h), and glutaraldehyde solution at a concentration of 4% was added slowly dropwise, and the stirring was continued for 1 h. The above mixture was centrifuged, and the purple precipitate was collected and washed with deionized water 3 times until the protein content of the washing solution was zero; finally, the purple precipitate was lyophilized to a constant weight, and PFL@ZIF-8@ZIF-67 immobilized lipase was obtained. 

### 3.3. Determination of Protein and Enzyme Activity

The protein content was determined using the Coomassie brilliant blue method [55], and bovine serum albumin (BSA) was used as the standard protein. Protein immobilization yield is defined as the amount of protein immobilized on the support as a percentage of the total amount of protein. Using p-NPB as substrate, the hydrolysis activity of the enzyme was determined by colorimetry. Dissolve 0.0195 g of p-NPB in 5 mL of acetone, add 1 mL of Triton-100, and make up to 25 mL with phosphate buffer (pH = 7.5, 0.2 M); 1 mL of the p-NPB solution and 14 mL of the phosphate buffer solution were measured, mixed in an Erlenmeyer flask, and pre-heated for 5 min at 40 °C; 20 mg of immobilized PFL or 3 mg of the free PEL was added to the reaction solution, and the reaction was carried out at 40 °C and 200 r/min for 5 min. The reaction was terminated by the addition of 1 M NaOH (5 mL) followed by centrifugation at 10,000 r/min for 10 min. Absorbance was recorded at 410 nm in a UV spectrophotometer (UV-6100, MAPADA, Shanghai, China) and compared with the standard curve of p-NP (end product of the enzymatic reaction). An enzyme activity unit is defined as the amount of p-NP produced per milligram of enzyme per hour. A specific activity is defined as the enzyme activity per milligram of protein.

### 3.4. Application of PFL@ZIF-8@ZIF-67 in Synthesis of Neryl Acetate

The transesterification activity of PFL@ZIF-8@ZIF-67 was investigated by using nerol and vinyl acetate to synthesize neryl acetate, and its reaction conditions were optimized. The specific experimental steps were as follows: in a closed Erlenmeyer flask, 2 mL of vinyl acetate was added, at which time the vinyl acetate acted as both reactant and solvent, and the concentration of nerol in the reaction system was ensured to be 100 mmol/L. The solution to be reacted was first preheated at 50 °C and 200 r/min for 10 min, then 28 mg of PFL@ZIF-8@ZIF-67 was added. Starting from the addition of PFL@ZIF-8@ZIF-67, 2 μL was taken out at intervals, and the content of neryl acetate was determined using gas chromatography. Ester exchange activity was defined as the amount of enzyme required to catalyze the production of 1 µmol neryl acetate per a minute.

### 3.5. Analysis Method of Neryl Acetate

The analysis of neryl acetate were performed using a GC apparatus that was equipped with a flame ionization detector and an SGE AC10 stainless steel column (Fuli, Taizhou, China). The area of the internal standard method was adopted, and hexadecane was used as the standard substance. The following specific test conditions were used: the N_2_ flow rate was 44 mL/min; the H_2_ flow rate was 40 mL/min; the air flow rate was 400 mL/min; the tail blowing rate was 25 mL/min; the split ratio was 10:1; the column temperature was kept at 165 °C; the injector temperature was 280 °C; and the detector temperature was 280 °C. The initial rate of the reaction is defined as the amount of neryl acetate generated per unit time and per unit volume.

### 3.6. Characterization 

The morphologies of PFL@ZIF-8@ZIF-67 were observed by scanning electron microscopy (SEM, SU-1510, Hitachi, Tokyo, Japan). The Fourier transform infrared (FT-IR) spectrum was scanned from 400 to 4000 cm^−1^ on a Nexus 670 instrument. X-ray diffraction (XRD) was carried out on an Ultima IV instrument (Tokyo, Japan).

### 3.7. Kinetics Analysis

The kinetics parameters of lipase and immobilized lipase samples were obtained by determining the initial reaction rates with p-NPB. The K_m_ value and V_max_ were calculated according to the Michaelis–Menten equation.

### 3.8. Reusability

The transesterification of nerol was used as a model reaction to investigate the reaction stability of lipase. First, 100 mmol/L of nerol was weighed in a 50 mL volumetric flask; then, the volume was fixed with vinyl acetate, and 3 mL was taken, respectively, in a stoppered flask. The free lipase (insoluble in vinyl acetate) or the immobilized lipase was added to the reaction at 50 °C, 200 r/min; the reaction time was 3 h, and the initial speed was measured by taking the solution 5 min before the reaction. After each reaction, the reaction solution was filtered out and the lipase was collected and reused.

### 3.9. Thermal Stability

A certain amount of the free PFL and PFL@ZIF-8@ZIF-67 was added to vinyl acetate (2 mL) preheated at different temperatures for 6 h; then, the solution was discarded and the preheated reaction solution (100 mmol/L nerol dissolved in vinyl acetate, 3 mL) was added to investigate the transesterification activity of the enzyme after heat treatment.

### 3.10. Storage Stability

The free PFL and PFL@ZIF-8@ZIF-67 were stored at room temperature in the conical flasks with a cover. The storage stability of the enzymes was investigated by taking a certain amount of enzyme weekly using a transesterification reaction system (100 mmol/L nerol dissolved in vinyl acetate, 3 mL) for the preparation of neryl acetate.

## 4. Conclusions

In conclusion, a new strategy was devised on MOF@MOF for the enhanced enzymatic performance of immobilized lipase. PFL@ZIF-8@ZIF-67 was successfully prepared and exhibited a significant increase in the specific activity. The specific activity of the immobilized enzyme was 20.4 times higher than that of the free enzyme under the optimized conditions. Meanwhile, PFL@ZIF-8@ZIF-67 can be effectively applied in the production of neryl acetate. The yield of neryl acetate reached 99% after 3 h of reaction and could still be maintained at 80% even after 10 cycles. More importantly, PFL@ZIF-8@ZIF-67 displayed better storage stability and thermal stability than the free lipase. This work provides a new outlook for biotechnological importance by immobilizing lipase on the ZIF-8@ZIF-67 and opens the door for its use in the industrial field.

## Data Availability

Raw data are available on request.

## Supplementary Materials

The following supporting information can be downloaded at https://www.mdpi.com/article/10.3390/molecules29122922/s1, Figure S1: Effect of immobilization time on the structure of immobilized lipase: (a) 2 h; (b) 2.5 h.
