# Supplementary figures and images for: Enhanced Enzymatic Performance of Immobilized Pseudomonas fluorescens Lipase on ZIF-8@ZIF-67 and Its Application to the Synthesis of Neryl Acetate with Transesterification Reaction
Source: Molecules. 2024 Jun 19;29(12):2922. doi: 10.3390/molecules29122922 (PMC11207022; doi:10.3390/molecules29122922)

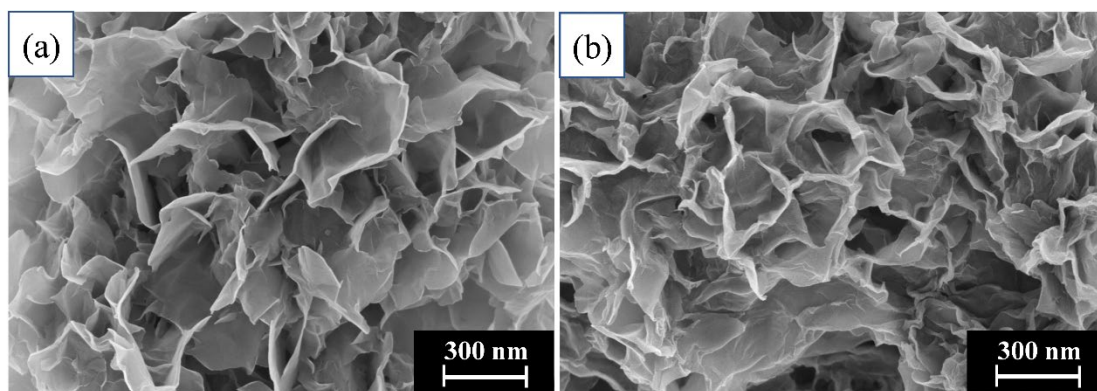

**Figure S1.** Effect of immobilization time on the structure of immobilized lipase: (a) 2 h; (b) 2.5 h.

Supplement: Supplementary file 1 [file molecules-29-02922-s001.zip › molecules-3054662-supplementary.pdf]
